# Phosphorus Concentrations in Sequentially Fractionated Soil Samples as Affected by Digestion Methods

**DOI:** 10.1038/srep17967

**Published:** 2015-12-09

**Authors:** Carlos A. C. do Nascimento, Paulo H. Pagliari, Djalma Schmitt, Zhongqi He, Heidi Waldrip

**Affiliations:** 1 Department of Soil Science, Luiz de Queiroz College of Agriculture, University of Sao Paulo, Av. Padua Dias, 11, Piracicaba SP, 13418-900, Brazil; 2 Department of Soil, Water, and Climate, University of Minnesota, Southwest Research and Outreach Center, 23669 130th St. Lamberton, MN 56152; 3 Santa Catarina State University, Lages (SC), Brazil; 4 USDA-ARS, Southern Regional Research Center, New Orleans, LA 70124; 5 USDA-ARS, Conservation and Production Research Laboratory, Bushland, TX 79012

## Abstract

Sequential fractionation has helped improving our understanding of the lability and bioavailability of P in soil. Nevertheless, there have been no reports on how manipulation of the different fractions prior to analyses affects the total P (TP) concentrations measured. This study investigated the effects of sample digestion, filtration, and acidification on the TP concentrations determined by ICP-OES in 20 soil samples. Total P in extracts were either determined without digestion by ICP-OES, or ICP-OES following block digestion, or autoclave digestion. The effects of sample filtration, and acidification on undigested alkaline extracts prior to ICP-OES were also evaluated. Results showed that, TP concentrations were greatest in the block-digested extracts, though the variability introduced by the block-digestion was the highest. Acidification of NaHCO_3_ extracts resulted in lower TP concentrations, while acidification of NaOH randomly increased or decreased TP concentrations. The precision observed with ICP-OES of undigested extracts suggests this should be the preferred method for TP determination in sequentially extracted samples. Thus, observations reported in this work would be helpful in appropriate sample handling for P determination, thereby improving the precision of P determination. The results are also useful for literature data comparison and discussion when there are differences in sample treatments.

Three decades ago, Hedley *et al.*^1^ developed a sequential extraction procedure as a tool to investigate changes in individual soil phosphorus (P) pools as affected by various management strategies. Since then, more than 1300 peer-reviewed studies have cited this sequential extraction procedure, and many others have adapted it for soils, manures, and other biological samples^2^,^3^,^4^,^5^. In brief, the original sequential extraction procedure consists of 0.5 g (dry weight; DM) of soil being extracted with resin in a given amount of solution (30 mL), followed by extraction of the soil residue with 30 mL of 0.5 M NaHCO_3_, 0.1 M NaOH, 0.1 M NaOH after sonication, and 1.0 M HCl. The remaining residue is then digested with a mixture of sulfuric acid (H_2_SO_4_) and hydrogen peroxide (H_2_O_2_) to determine residual P^1^. The fractions were functionally defined as follows: soluble (labile) inorganic P (P_i_) is extracted in the resin and NaHCO_3_ fractions; P_i_ associated with Al and Fe is extracted with NaOH; P_i_ occluded in the interiors of amorphous Al/Fe hydrous oxides is extracted with NaOH and sonication; P_i_ associated with Ca and primary minerals is extracted with HCl; and the most stable organic P (P_o_) and occluded P_i_ are estimated after the H_2_SO_4_/H_2_O_2_ digestion of the residue^1^,^6^. Organic P (P_o_) concentrations are then estimated for each fraction as the difference in P_i_ detected by colorimetric procedures, such as the Murphy and Riley^7^ molybdate blue method, before and after digestion of P_o_ to P_i_^1^.

The final calculated concentration of P_o_ in sequentially fractionated samples is, in most instances, affected by several factors, including the pore size of filters used to remove suspended materials in the extract and degree of oxidation of organophosphates during the digestion procedure. Although the original method as described by Hedley *et al.*^1^ does not include filtration, many have adapted the method to incorporate the use of a 0.45 μm pore-size nitrocellulose acetate filter for soil extracts^5^,^8^,^9^ and biological samples^8^,^10^,^11^. For the digestion of several different extracts, some have used potassium persulfate (K_2_S_2_O_8_) and H_2_SO_4_^12^,^13^,^14^,^15^,^16^, or concentrated H_2_SO_4_ in combination with H_2_O_2_ for soil samples^5^,^12^ and for biological samples^3^,^8^,^12^. Other researchers have used perchloric acid (HClO_4_) for digestion^13^,^17^. However, Pierzynski^18^ indicated that laboratory digestion procedures were not capable of completely oxidizing soil organic compounds and liberating P_i_ into solution, thereby underestimating P_o_ concentrations in a sample. Indeed, total P (TP) determined after digestion was even lower than P_i_ determined without digestion in some cases^14^,^19^. In addition, the detection of P_o_ can be influenced by the method of P_i_ determination due to hydrolysis of P_o_ or solubilization of condensed P_i_ by the reagents used during color development in molybdate blue colorimetric methods^20^.

The most important criteria for recommending digestion and quantification methods for P determination in soils are accuracy, simplicity, and rapidity of determination^21^. The use of inductively coupled plasma optical emission spectroscopy (ICP-OES) is becoming increasingly prevalent for determination of TP in aqueous samples, including soil extracts. The inductively coupled plasma from ICP-OES causes atom and ion excitation with emission of electromagnetic radiation at wavelengths characteristic of specific elements^22^. As all compounds in a sample solution are decomposed to elemental plasma statuses, ICP-OES determines TP (i.e., both P_i_ and P_o_) in a sample without the need for prior digestion^22^,^23^,^24^. The calculated difference in P content of a sample evaluated by both ICP-OES and a colorimetric procedure to determine P_i_ is generally considered to represent P_o_. Numerous papers have been published to compare the difference in soil test P levels determined by colorimetric and ICP methods^21^,^23^,^25^,^26^. In general, the P concentrations measured with ICP-OES are greater than those obtained following colorimetric procedures, reflecting the amount of P_o_ in the samples^21^,^23^,^25^,^26^. However, to our knowledge, there have been no studies that reported or compared the concentrations of TP in sequentially extracted soil samples as determined by ICP-OES following different digestion methods as well as in undigested extracts.

This study was conducted to compare P concentrations in sequentially extracted solutions following digestion by autoclaving with H_2_SO_4_ and K_2_S_2_O_8_, heated digestion with H_2_SO_4_ and H_2_O_2,_ and direct determination of undigested extracts with ICP-OES. In addition, the effects of sample filtration (all fractions) and acidification (NaHCO_3_ and NaOH fractions) were evaluated. Acidification of sequential extracts has been performed for two reasons: (1) the phosphatase hydrolysis method of He and Honeycutt (2001) for functional identification of specific organic P forms requires a standardized pH, and (2) ICP-OES analyses of alkaline samples could lead to metal precipitation in the aerosol chamber of the ICP instrument, which is avoided by using acidified samples^27^. The current study was conducted with 20 soil series collected from Brazil and four states across the U.S.: Minnesota, Iowa, Wisconsin, and Texas. Figure 1 shows a detailed description of the sequential fractionation and sample manipulation used in this study. Our hypothesis was that no significant differences would be found in TP concentration determined in extracts between the digestion methods and undigested analysis using ICP-OES.

## Results and Discussion

### Effects of digestion method and filtration on measured water-extractable P

Total water-extractable P concentrations of soils measured with ICP-OES varied greatly depending on which digestion method was used, the specific soil series, and whether the samples were filtered or not prior to digestion (Table1). The block digestion method resulted in the highest measured TP concentrations in most water extracts, while most of the undigested water-extracts had lower measured TP concentrations. The general trend was that when the P concentration in the undigested sample was greater than 14.0 mg kg^−1^ there were no significant (*P* > 0.05) differences in TP concentrations among digestion methods, with the exception of the Clarion soil (Table 1). The ratio of TP concentration determined with the block and autoclave methods, with respect to the undigested method, was calculated by dividing the block P concentration and the autoclave P concentration by the undigested P concentration determined by ICP-OES (Fig. 2). There is a clear non-linear relationship between the P concentrations measured following block digestion and those measured in undigested water extracts (Fig. 2). The non-linear regression shows that the ratio between measured P concentrations in block digested and undigested extracts become one at 17.9 mg kg^−1^ (value calculated by solving the equation 1.0 = 10.4 * *X* ^ - 0.8 reported in Fig. 2). This non-linear relationship was not observed for the autoclave digested extracts when compared to undigested extracts, likely due to high variability when the measured TP concentrations in undigested extracts were below 17.9 mg kg^−1^. Similar results were found by Matula^28^ while investigating the effect of digestion (block digestion with K_2_S_2_O_8_) on water-soluble P from soil samples. The authors reported that at low water-soluble P concentrations, the digestion method overestimated the amount of P in solution and the relationship followed an exponential function^28^. Vadas and Kleinman^29^ also reported that digested water extracts of manure samples consistently had higher TP concentration compared with undigested samples. Although no direct cause has been determined for the fact that TP concentration in digested samples containing low P concentrations, it is possible that differences in the sample matrix could be involved. For example, de Boer *et al.*^30^ reported that the presence of K, Mg, and Na could interfere with P determination when using ICP for determination of total P water samples.

Filtration was found to be a bigger issue with the autoclave method than the block method or undigested water extracts (Table 1). For samples digested using the autoclave method, 50% of the soils showed a significant (*P* < 0.05) filtration effect, while only one and three of the soils were affected by filtration for the block digested and undigested samples, respectively (Table 1). Filtering the samples prior to digestion, in most cases, caused a significant decrease in the TP concentration, with the exception of the Brazil and Clarion soils. Although the results are not well understood, it is possible that filtration through a pore size of 0.45 μm prohibited the inclusion of large organophosphates, other organic-P complexes, and some mineral-associated P colloids^31^. Correlation analyses were performed to try and identify if any of the soil properties presented in Table 2 could be affecting filtration for the soils that showed a significant filtration effect, in particular regarding the clay distribution of the soils. We observed no significant correlations between the ratio of block digestion divided by undigested samples and autoclave digestion divided by undigested samples. However, the lack of consistency on the effect of filtration among the digestion methods makes it difficult to draw final conclusions regarding the effect of filtration on the water fraction as in some cases there was an increase in the TP measured.

Overall, it appears that both the block and autoclave digestion methods tended to overestimate the amount of water-extractable TP in a sample, particularly at low P concentrations. Analyses of water extractable P with ICP-OES, without digestion or filtration, decreased variability as compared to block and autoclave digestion or on extracts that had undergone filtration.

### Effects of digestion method and filtration on measured NaHCO_3_-extractable P

The concentrations of TP in the NaHCO_3_ fraction changed significantly (*P* < 0.05) based on specific soil series, digestion method, filtration, and acidification (Tables 3 and 4). In contrast to results observed for water extracts, measured TP concentrations were generally lowest with block digestion and highest following autoclave digestion. Filtering the NaHCO_3_ extracts caused a significant (*P* < 0.05) reduction in TP soluble in NaHCO_3_ in 15 of the 20 soils following digestion with the autoclave method and in three soils following digestion with the block method. In contrast, as compared to undigested NaHCO_3_ extracts, filtration caused an increase in measured total NaHCO_3_-soluble P for the Estherville soil, autoclave digested Hubbard soil, and four soils digested with the block method (Table 3). As observed for water extracts, filtration likely retained large P-containing organic and inorganic compounds. Turner and Haygarth^32^ reported that from 21% to 46% of TP in leachate was retained by filtration at 0.45 μm. In the present study, the amount of NaHCO_3_-P that was presumably retained by filtration ranged between 15% and 57% for the autoclave and block methods. As observed with water extracts, filtration did not cause significant (*P* > 0.05) changes in TP measured in undigested samples, with the exception of the Estherville soil. The lack of other studies that compared filtration and digestion methods makes it difficult to draw conclusions or hypothesize the rationale for increased TP concentration measured after filtration.

Acidification of the NaHCO_3_ extracts, in most cases, caused a significant (*P*  < 0.05) reduction in TP concentrations (Table 4). For the unfiltered samples, acidification caused a TP concentration reduction in 13 of the 20 soils tested and increased TP concentration in the Barnes soil. For the filtered samples, there was a decrease in TP concentration in 7 of the 20 NaHCO_3_ extracts. It is possible that acidification of this fraction caused precipitation of P_o_ and/or Al/Fe-associated P, which led to reduced concentrations of NaHCO_3_-P. As an example, soil humic acids are typically purified by acidification, where high molecular weight humic acids are precipitated from solution with 1.0 M HCl^33^.

### Effects of digestion method, dilution and filtration on measured NaOH-extractable P

High concentrations of OM in NaOH extracts made it necessary to dilute this fraction prior to filtration in order to avoid clogging of membrane filter pores. Therefore, the treatments for this fraction were: unfiltered/undiluted, unfiltered/diluted, and filtered/diluted NaOH extracts. As observed for the other fractions, most of the significant differences (*P* < 0.05) were observed with the block and autoclave digestion methods; while the least amount of variability was observed in the undigested samples. Figure 3 presents the distribution of the NaOH fraction for unfiltered/undiluted, unfiltered/diluted, and filtered/diluted samples analyzed using all three methods: undigested extracts analyzed directly with ICP-OES (Fig. 3A), block digestion (Fig. 3B), and autoclave digestion (Fig. 3C). It is clear from the *R*^2^ values that analyzing the NaOH extract directly with ICP-OES (no digestion) produced the least variable results (*R*^2^ = 0.99, unfiltered; *R*^2^ = 0.97, filtered) (Fig. 3A–C). Diluting samples prior to analyses caused a significant (*P* < 0.05) change in total NaOH-P concentrations in 6 out of 20 (block digestion) and 15 out of 20 (autoclave digestion) soils, with no significant changes in P concentration for undigested NaOH extracts. In 5 of the 6 block digested NaOH extracted soils, there were decreased measured TP concentrations after dilution, decreases which ranged between 49.9 to 105.7 mg P kg^−1^. However, one soil showed an increase (*P* < 0.05) in measured P concentration of 19.5 mg P kg^−1^ (Fig. 3B). Whereas, for the autoclave method, TP significantly (*P* < 0.05) increased after dilution in 6 soils and decreased in 9 soils (Fig. 3C). There were no significant (*P* > 0.05) differences due to dilution in undigested NaOH extracts (Fig. 3A).

The effects of filtration on measured NaOH-P were conflicting and depended on the digestion method. In some cases, filtration caused a decrease in measured NaOH-P concentrations, but in others it caused an increase for both block and autoclave digested extracts (Fig. 3A–C). As observed for the water and NaHCO_3_ fractions, both digestion methods introduced a good deal of variability in measured NaOH-P as indicated by the number of significant differences due to filtration (27 out of 40 samples for both digestion methods combined), while undigested extracts showed very little variation. For the undigested method, only the Mt. Carroll NaOH extract had a significant (*P* < 0.05) increase of 13.8 mg P kg^−1^ in TP concentration after filtration. Filtration played a more important role in block and autoclave digested extracts. Block digested NaOH extracts from 7 soils showed an increase in measured TP after filtration, and 6 soils had decreased NaOH-P levels after filtration. For the autoclave method, filtration caused an increase in total NaOH-P levels in 6 soils and a reduction in 8 soils. As observed for NaHCO_3_-P, the reduction in measured P following filtration could be attributed to retention of large organic or mineral compounds that contain P, or clogging of pores could have kept small organic compounds from passing through the filter. However, the rationale for the observed increase in TP concentration in some filtered samples cannot be readily elucidated. The variation added by filtration in some cases exceeded 50%. For example, the Barnes soil that was unfiltered and undiluted had a measured NaOH-P concentration of 68.5 mg kg^−1^ following autoclave digestion, while the diluted, unfiltered NaOH extract contained 138.3 mg P kg^−1^.

Acidification of the NaOH extracts caused a significant (*P* < 0.05) change in TP concentrations measured in almost all soils; however the effect of acidification was confounded with the filtration and dilution effects (Table 5). Although there was a significant acidification effect, there was no consistent trend of whether measured NaOH-P concentrations increased or decreased after acidification. There were no significant (*P* > 0.05) changes in measured TP concentration in the Barnes soil. However, there were contradicting results observed for some soils. For example, acidification effects were not significant for undiluted Estherville NaOH-P, but acidification increased (*P* < 0.05) measured TP in diluted/unfiltered extracts by 70.5 mg P kg^−1^, and decreased (*P* < 0.05) TP in filtered/diluted extracts by 15.7 mg P kg^−1^. Similar results where the effects of acidification were dependent on filtration and dilution were also observed for the Normania and Storden soils. There were no soil properties measured among the soils studied that would suggest a possible explanation for the observed results, as no correlation were detected. For the remaining soils, acidification would either significantly (*P* < 0.05) increase or decrease TP concentrations determined in the extracts. However, none of the soil properties measured were useful in trying to understand the nature of the behavior observed for the effect of acidification. As previously mentioned for NaHCO_3_ extracts, it could be due to flocculation or precipitation of acid-insoluble inorganic and organic P-containing compounds. However, it is not known why acidification would increase TP determined in the samples under some conditions but not others.

### Effects of digestion method and filtration on measured HCl-extractable P

The amount of P measured in the HCl fraction was significantly (*P* < 0.05) affected by the digestion method, filtration, and their interaction (Table 6). In many cases (9 out of 20 extracts), unfiltered/block digestion treatment resulted in the highest measured HCl-P concentrations, followed by unfiltered/autoclave digested and unfiltered/undigested HCl extracts. However, there were cases where either the undigested or autoclave unfiltered samples were the highest, showing an inconsistent trend (Table 6).

Filtration of HCl extracts was a major issue for the block and autoclave digestion methods, but less so for undigested extracts (Table 6). Nineteen of the extracts analyzed by ICP-OES after block digestion had decreased HCl-P if filtered prior to digestion. One exception was the Storden soil, which showed a significant (*P* < 0.05) increase P concentration after filtration. The reduction in TP after filtration of the block digested samples ranged between 8% and 80%. For the autoclave method, 9 soils showed decreased TP after filtration, decreases which ranged between 6.8% and 28%, and 6 soils showed an increase in TP after filtration, ranging between 5% and 43%. For the undigested HCl extracts, there were decreased TP concentrations for only three soils: the reduction ranged between 8% and 14%. The significant (*P* < 0.05) filtration effect for both block and autoclave digested HCl-P suggests that the P_o_ extracted in this fraction was likely associated with large organic moieties. However, the lack of a similar trend in the undigested HCl extracts precludes a thorough understanding of the mechanisms behind decreased measured TP in filtered, undigested HCl extracts. Until recently the HCl fraction was believed to predominantly contain inorganic-P forms of P (e.g., Ca-phosphates). However, He *et al.*^20^ showed that a significant amount of P_o_ is present in HCl extracts of soils and manures: in some cases, HCl-P_o_ concentrations were higher than HCl-P_i_.

### Implications of this work’s findings

During sequential fractionation studies it is mostly common to partition the P extracted from each fractions into P_i_ and P_o_. The amount of P_o_ in each extract is estimated as the difference between the TP determined in a sample minus the P_i_ determined in the same sample. Therefore, when the TP portion is wrongly determined, the P_o_ in that samples will automatically be wrongly calculated.

In this study, the significant effect of digestion and filtration on measured TP in the sequential extracts, led to significant effects of digestion and filtration in the total P_o_ determined in the soils studied (Table 7). The effect of filtration was significant (*P* < 0.05) and the magnitude of the changes in total P_o_ determined varied by digestion method used. For the undigested samples, filtration caused a reduction in total P_o_ determined in 4 samples and the decrease ranged between 9 to 16%; while there was an increase of 11% in total P_o_ measured after filtration for the Hubbard soil (Table 7). For the block digested samples, filtration caused a reduction in calculated P_o_ concentration of 10 soils, which ranged between 19 and 71%, and increased the calculated P_o_ concentration in three soils, increase which ranged between 11 and 112%. For autoclave digested extracts, filtration caused a reduction in calculated P_o_ that ranged between 11 and 70% in 10 soils, and increases of 26 and 34% for the Seaton and Normania soils, respectively. As observed for the other fractions, filtration would be expected to decrease concentrations of calculated P_o_; however, the rationale behind increased P_o_ after filtration is difficult to explain. Other researchers have also reported increased TP concentration determined in digested filtered solutions. For example, McDowell and Sharpley^34^ and Vadas *et al.*^35^ reported 13% increase in TP concentration in filtered digested samples and Sharpley and Moyer^36^ reported up to 24% increases in TP concentration in digested filtered samples.

The results of this study showed that sample manipulation after sequential fractionation of soil samples can strongly effect TP concentration measured by ICP-OES. Some of the factors contributing to the observed differences in TP measured after digestion are incomplete oxidation, which depends on concentration of oxidant, digestion temperature, sample matrix, soil type and organic matter content, and hydrolysis of organically bound P during analysis^37^. The least variability in measured TP was obtained by direct ICP-OES analysis without digestion, filtration, or acidification of soil extracts; whereas, either block or autoclave digestion increased data variability. Dilution or filtration of block and autoclave digested extracts were sources of variation, further indicating that direct ICP-OES analysis of undigested extracts is the preferred method to minimize data variability. As a result of the high data variability with digestion, the amount of calculated P_o_ in the extracts also varied greatly. We believe observations reported in this work are useful and suggest appropriate handling of soil and other environmental (e.g. runoff water, leachate, lake water, and so forth) samples for P determination, thus improving the precision of P determination. These results are also helpful for data interpretation and discussion when there are differences in sample treatments among different studies. Therefore, we recommend direct ICP-OES, without extract manipulation, for future research with sequential fractionation of soil or other environmental samples, such as lake sediment extracts, runoff rain water, drainage water, and others.

## Materials and Methods

### Soil sample collection and sequential fractionation

Soil samples were collected from Brazil (an Ultisol known as Red-Yellow Argisol, hereinafter Brazil) and across the U.S., including soils from Minnesota Barnes loam, Clarion loam, Cordova clay loam, Fargo silty clay, Formdale clay loam, Hubbard loamy sand, Lester loam, Mount Carroll silt loam, Nicollet clay loam, Normania loam, Seaton silt loam, Storden loam, Walter sandy loam, and Zimmerman sand; the Texas Panhandle Amarillo sandy loam, Pullman clay loam, and Randall clay; Iowa Estherville sandy loam; and Wisconsin Pella clay loam. Most of the soils were from cropped fields under conventional agricultural practices, but had differing histories of cropping system, management, and fertilizer use, with the exception of the Randall series. The Randall clay soil is not typically cropped, as it has high clay content (~38 to 42%), very low permeability, and extensive cracking when dry. The Randall clay used in this study was collected from the bottom of a playa basin in Bushland, Texas. All soils samples were collected from 0 to 15 cm to represent the depth most affected by tillage operations.

After collection, soils were sieved (2 mm), air-dried, and stored at room temperature (22 °C) until analyses. The use of soils encompassing a wide range of origins, textures, and chemical compositions should allow for broader inferences regarding the concentrations of measured P as a function of digestion method, filtration, and acidification. Soil pH was measured in water (1:1 ratio w/w). Organic matter (OM) content was measured by loss on ignition at 360 °C, and particle size analyses were performed using the hydrometer method of Bouyoucos^38^. Soil test P was extracted with the Bray-1 and Olsen reagents^39^ and determined by the molybdate blue method of Murphy and Riley^7^. Calcium (Ca^2+^), magnesium (Mg^2+^), iron (Fe^3+^), and aluminum (Al^3+^) were extracted using Mehlich-3^39^ and determined by ICP-OES (PerkinElmer, Optima 8 × 00, Norwalk, CT). Selected soil properties are presented in Table 1.

For the sequential fractionation, the original procedure of Hedley *et al.*^1^ was used with the modifications of He *et al.*^20^: soil mass was increased from 0.5 g to 2.0 g and extraction solution volume was decreased from 25 mL to 20 mL; distilled water was used for extraction of labile P, rather than resin; and the 0.1 M NaOH extraction following ultra-sonication step was omitted. Soil samples (2.0 g dry weight, four replications per sample) were sequentially extracted with deionized water (20 mL) for 16 h at 22 °C on an orbital shaker (250 rpm). Extracts were then centrifuged at 3,400 × *g* for 15 min at 4 °C and supernatants were carefully decanted into clean 50 mL tubes. Soil residues retained in the tubes were then sequentially extracted with 20 mL each of 0.5 M NaHCO_3_ (pH 8.5), 0.1 M NaOH, and 1.0 M HCl for 16 h, with extraction conditions and supernatant collection as previously described.

### Manipulation of sequential fractions: filtration, digestion, dilution and P determination

**Figure 1:**
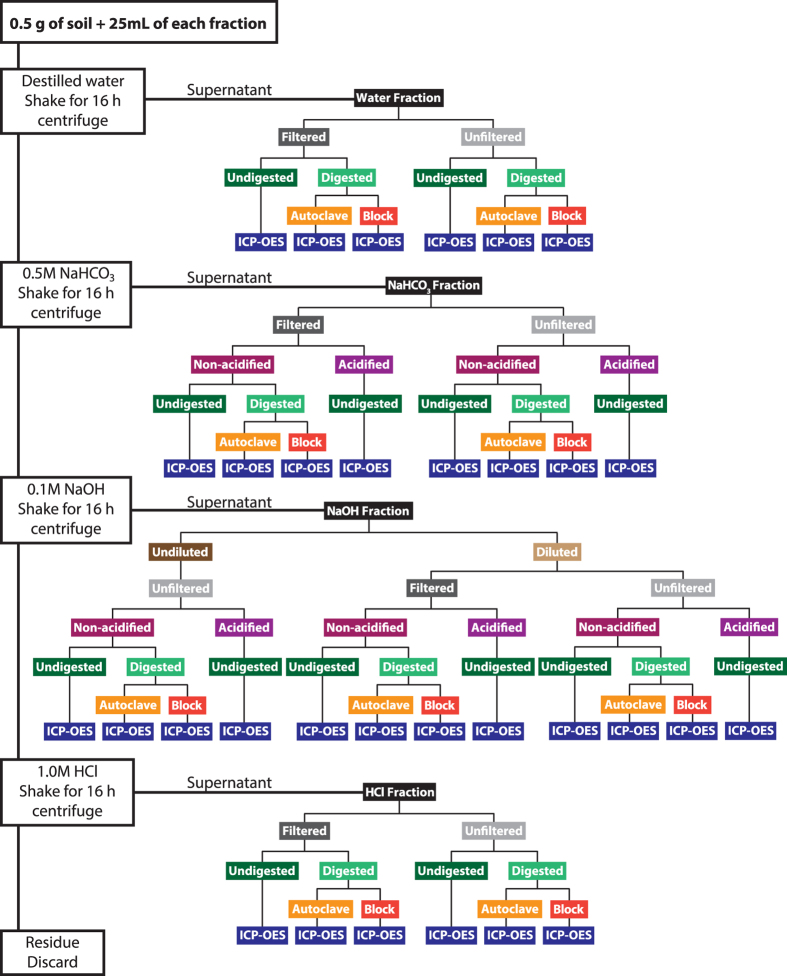
Visual representation of sequential fractionation and post-extraction manipulation and treatments of extracts.

**Figure 2:**
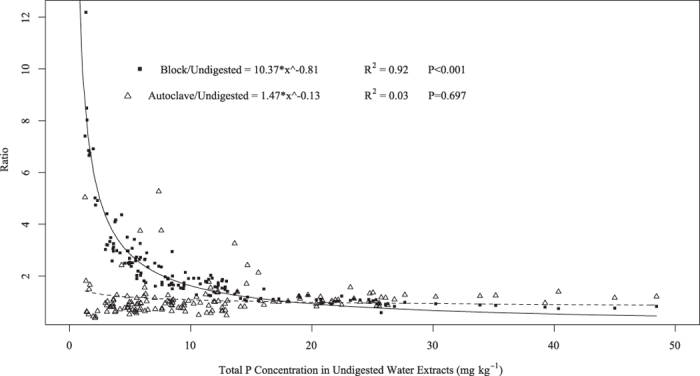
Ratio between total P concentration determined by the block method (black squares) and autoclave method (open triangle) in relation to the total P concentration determined by the undigested method.

**Figure 3:**
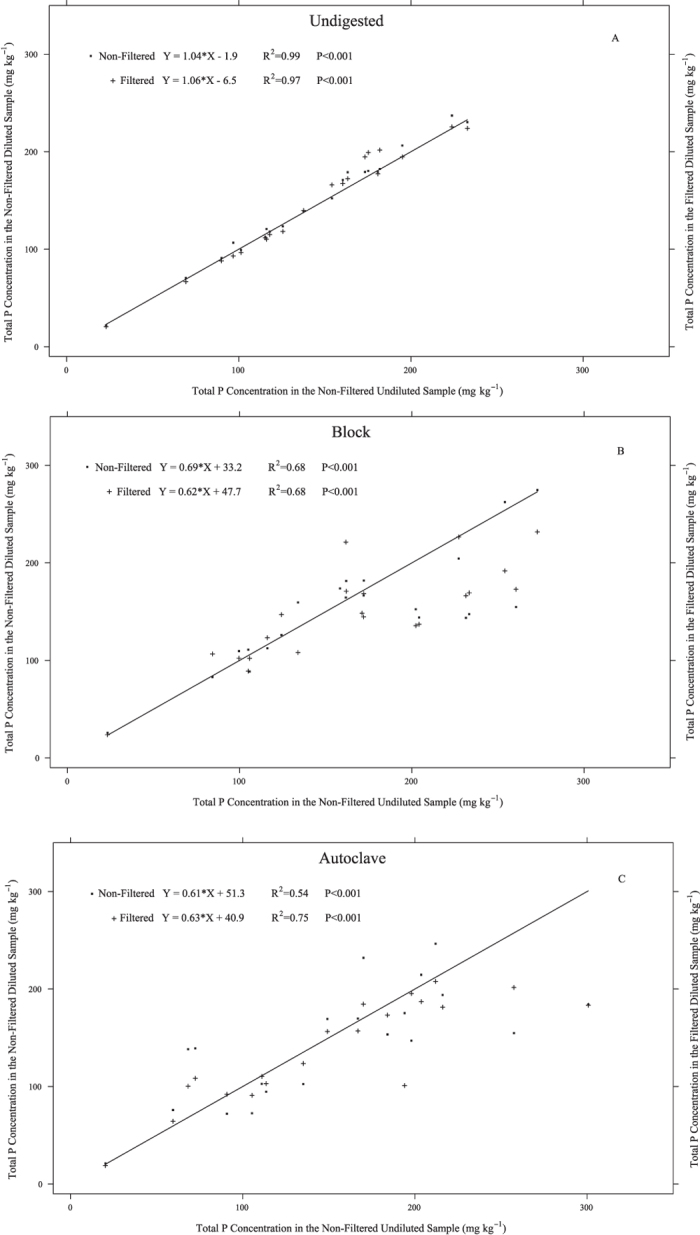
Relationship between total P concentrations in NaOH extracts for the undiluted unfiltered samples plotted against the diluted unfiltered (full square) and diluted filtered (plus sign) for the undigested (A), block (B), and autoclave (C) methods. Solid line in all panels is the 1:1 relationship.

After sequential extraction, the water and HCl fractions were separated into two sub-fractions: (1) unfiltered and (2) filtered (0.45-μm nitrocellulose acetate membrane). The NaHCO_3_ extracts were separated into four sub-fractions: (1) unfiltered and non-acidified, (2) unfiltered and acidified, where NaHCO_3_ extracts were diluted and pH adjusted with 6 M HCl, (3) filtered and non-acidified, and (4) filtered and acidified. The NaOH extracts were separated into six sub-fractions based on pH and filtration interference by OM. High concentrations of OM were solubilized during NaOH extraction tended to clog filter pores; thus, extracts were diluted 1:10 with deionized water. Some NaOH sub-fractions were acidified with 1 M HCl. As a result, the NaOH extract was separated into six sub-fractions: (1) non-diluted, unfiltered, non-acidified, (2) non-diluted, unfiltered, acidified, (3) diluted, unfiltered, non-acidified, (4) diluted, unfiltered, acidified, (5) diluted, filtered, non-acidified, and (6) diluted, filtered, acidified. For the purpose of this research, acidification of samples was done primarily because ICP-OES analyses are commonly conducted on acidified samples. Samples were acidified to pH ranging between 0.0 and 1.0. There was no observed precipitation of soil minerals, however, there was precipitation of OM complexes following acidification of many of the NaHCO_3_ and NaOH samples. A schematic diagram of extract handling procedures is presented in Fig. 1. There were a total of 14 treatments conducted on each soil sample.

Two methods were used for sample digestion of all sub-fractions, with the exception of the acidified NaHCO_3_ and NaOH extracts that were analyzed directly with ICP-OES. The digestion methods were: (1) concentrated H_2_SO_4_ + 7.5% K_2_S_2_O_8_, where 2 mL of samples were mixed with 1 mL of H_2_SO_4_ and 10 mL of a 7.5% K_2_S_2_O_8_ and autoclaved at 121 °C for 2 h at 1 atm (hereinafter “autoclave” method) (EPA ESS method 310.2); and (2) concentrated H_2_SO_4_ + H_2_O_2_, where 2 mL of each extract was mixed with 2 mL of concentrated H_2_SO_4_ and heated to 225 °C, with additions 1 mL H_2_O_2_ at 10 min intervals until the digestate was clear (hereinafter referred to as “block”) (Hedley *et al.*^1^). For comparison, all extracts were also directly analyzed with ICP-OES (hereinafter referred to as “undigested”). For quality control, blanks and controls were included in the analyses, where soils were omitted and the extracting solutions were digested to assure the absence of contamination and correct for background. In addition, extraction solutions with known amounts of TP were prepared and treated as samples during digestion and subsequent P determination to assure no loss of P occurred during the digestion and that ICP-OES results were valid.

For P determination, P_i_ was determined in all undigested samples using the molybdate blue method as described by He and Honeycutt^40^ using a Biotek Epoch microplate spectrophotometer (Biotek, Winooski, VT). TP was determined using an ICP-OES.

TP in a sample is the sum of P_i_ and P_o_ and can be described as:


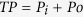


however, during ICP-OES analysis both, the P_i_ and P_o_ are analyzed as P_i_ as described earlier.

### Statistical analyses

The effect of sample filtration, extract acidification, dilution, digestion method, and their interactions (when appropriate, e.g. HCl and water fractions were not acidified and therefore no interaction existed) were evaluated using repeated measures analyses with Proc Glimmix in SAS 9.3^41^,^42^. The Akaike information criteria (AIC) value was used as the model selection criteria to determine the best covariance model for the repeated variable. Significance of differences among the sample manipulation methods (*P* < 0.05) were determined by mean separation using Fisher’s least significance difference test (LSD). Regression analysis and correlation analysis were performed using R^43^. All data analyses were performed on replicate data, while the results are presented as the average of four replicates. The dataset was analyzed for the presence of outliers before any statistical test was conducted.

## Additional Information

**How to cite this article**: do Nascimento, C. A. C. *et al.* Phosphorus Concentrations in Sequentially Fractionated Soil Samples as Affected by Digestion Methods. *Sci. Rep.* **5**, 17967; doi: 10.1038/srep17967 (2015).

## Figures and Tables

**Table 1:** Average total P concentration in sequentially extracted soil samples as determined in undigested, block digestion, and autoclave digestion methods in the water fraction.

Soil Series	Filtered	Extract		Block Digestion		Auto Clave	
		mg kg^−1^					
Amarillo	No	3.8	c^†^	11.4	a	6.2	b
	Yes	3.7	c	11.1	a	3.5	b
Barnes	No	0.7	b	14.0	a	3.7	b
	Yes	1.4	b	12.1	a	1.1	b
Brazil	No	6.3	b	18.3	a	1.7	c
	Yes	6.1	b	17.2	a	5.1	b
Clarion	No	14.6	bc	16.6	ab	6.5	d
	Yes	12.5	c	19.1	a	12.6	c
Cordova	No	11.3	a	15.9	a	9.1	a
	Yes	9.7	a	16.0	a	10.3	a
Estherville	No	8.3	c	13.6	a	8.4	bc
	Yes	6.0	c	11.3	ab	5.2	c
Fargo	No	22.9	a	20.8	a	24.1	a
	Yes	24.2	a	25.1	a	19.0	a
Formdale	No	14.3	ab	19.6	a	18.1	a
	Yes	12.4	b	20.0	a	9.1	b
Hubbard	No	10.8	c	20.9	a	18.6	a
	Yes	8.8	d	16.2	b	7.7	d
Lester	No	12.4	b	21.7	a	18.8	a
	Yes	9.8	c	20.4	a	10.4	bc
MtCarrol	No	2.2	bc	10.7	a	5.1	b
	Yes	1.3	c	10.8	a	2.1	bc
Nicollet	No	5.5	bc	16.3	a	7.9	b
	Yes	2.2	c	14.5	a	5.1	bc
Normania	No	4.6	c	12.7	a	6.4	b
	Yes	3.5	c	12.5	a	4.8	c
Pella	No	21.2	a	22.2	a	22.1	a
	Yes	17.0	a	18.5	a	16.7	a
Pullman	No	43.3	a	33.8	bc	37.3	ab
	Yes	31.7	bc	28.9	bc	25.8	c
Randall	No	24.0	a	27.0	a	23.9	a
	Yes	19.6	a	23.3	a	16.3	a
Seaton	No	6.6	b	14.6	a	12.1	a
	Yes	5.1	b	12.2	a	5.4	b
Storden	No	5.1	b	13.2	a	8.3	ab
	Yes	3.2	b	9.5	a	3.5	b
Walter	No	25.7	ab	23.7	b	29.5	a
	Yes	21.2	b	19.7	b	21.1	b
Zimmerman	No	7.9	bc	12.9	a	10.8	ab
	Yes	5.0	bc	11.6	a	6.1	c

^†^Means for a soil series followed by the different letter are significantly different (P < 0.05).

**Table 2:** Summary of selected soil properties.

Soil Series	Olsen P	Bray-1 P	Ca†	Mg	Fe	Al	Sand	Clay	OM	pH
	mg kg^−1^						%			
Amarillo	3	8	1538	248	38	491	72.2	13.5	1.6	7.6
Barnes	7	16	3815	526	48	32	22.7	22.4	5.5	8.0
Brazil	2	8	451	137	43	702	78.2	20.1	2.8	5.3
Clarion	44	75	2770	593	201	586	36.2	38.4	4.4	6.2
Cordova	35	46	4899	566	82	11	17.4	29.6	8.9	7.5
Estherville	16	44	3084	536	118	259	60.1	22.7	3.9	7.0
Fargo	42	87	3606	1410	47	17	5.2	6.7	6.8	7.7
Formdale	31	51	3480	816	46	10	29.4	21.0	6.9	7.6
Hubbard	20	82	580	95	95	716	82.5	11.8	1.3	6.6
Lester	21	25	2645	425	106	278	40.2	32.1	5.1	6.6
Mt Carroll	13	22	2829	321	83	134	15.3	16.0	4.5	7.9
Nicollet	17	9	2757	647	120	347	36.1	35.1	4.4	6.7
Normania	23	44	2414	608	231	703	37.0	30.9	4.4	5.5
Pella	37	96	691	166	185	633	26.4	22.6	5.6	7.7
Pullman	52	68	2953	414	40	144	31.4	34.3	2.7	7.2
Randall	40	74	4725	569	115	362	30.1	40.3	3.4	7.8
Seaton	34	39	2046	425	149	442	7.4	25.9	3.4	6.7
Storden	25	42	1995	349	162	656	43.3	30.9	4.8	5.5
Wheatville	22	24	3062	925	20	0	19.3	11.5	4.7	8.3
Zimmerman	15	38	1561	60	211	368	81.0	14.3	1.9	8.0

^†^Ca, Mehlich-3 extractable calcium; Mg, Mehlich-3 extractable magnesium; Fe, Mehlich-3 extractable iron; Al, Mehlich-3 extractable aluminum.

**Table 3:** Total P concentration in the sequentially extracted NaHCO_3_ fraction of soil samples determined by ICP-OES after undigested, block digestion, and autoclave digestion treatments.

Soil Series	Filtered	Undigested		Block digestion		Autoclave digestion	
		mg kg^−1^					
Amarillo	No	8.8	b†	9.5	b	17.0	a
	Yes	10.0	b	9.8	b	7.3	b
Barnes	No	16.8	b	20.5	b	17.8	b
	Yes	18.0	b	28.3	a	15.8	b
Brazil	No	11.5	c	17.8	a	15.3	b
	Yes	11.8	c	17.8	a	11.8	c
Clarion	No	71.0	b	69.5	b	79.8	a
	Yes	70.0	b	58.0	d	63.8	c
Cordova	No	61.3	b	60.3	b	72.5	a
	Yes	61.0	b	51.5	c	50.3	c
Estherville	No	43.5	c	33.5	d	50.3	a
	Yes	48.0	b	41.5	c	42.8	c
Fargo	No	74.3	ab	62.0	b	82.8	a
	Yes	74.0	ab	65.5	ab	61.8	b
Formdale	No	45.5	b	39.3	c	45.8	b
	Yes	45.0	b	51.0	a	43.5	bc
Hubbard	No	39.3	b	23.0	d	36.0	c
	Yes	39.0	b	39.8	b	42.8	a
Lester	No	42.8	a	38.3	b	38.0	b
	Yes	43.0	a	40.5	ab	40.5	ab
MtCarrol	No	35.3	bc	32.5	c	39.5	a
	Yes	36.8	ab	34.8	bc	31.8	c
Nicollet	No	36.8	a	34.5	a	36.3	a
	Yes	37.5	a	35.0	a	34.0	a
Normania	No	42.8	b	39.3	c	48.0	a
	Yes	42.8	b	38.5	c	39.3	c
Pella	No	64.5	b	49.3	c	74.0	a
	Yes	67.8	b	49.3	c	48.8	c
Pullman	No	108.3	b	58.3	d	142.0	a
	Yes	110.5	b	24.5	e	95.8	c
Randall	No	81.8	b	51.8	c	95.8	a
	Yes	88.5	ab	49.5	c	78.3	b
Seaton	No	49.8	ab	47.0	bc	52.5	a
	Yes	50.8	ab	43.0	c	44.8	c
Storden	No	28.8	bc	23.0	c	41.8	a
	Yes	32.0	b	20.5	c	25.3	c
Walter	No	85.0	b	59.5	d	92.8	a
	Yes	83.8	b	63.5	d	77.3	c
Zimmerman	No	32.0	ab	29.8	bc	36.0	a
	Yes	31.5	bc	28.0	bc	27.0	c

^†^Means for a soil series followed by the different letter are significantly different (*P* < 0.05).

**Table 4:**
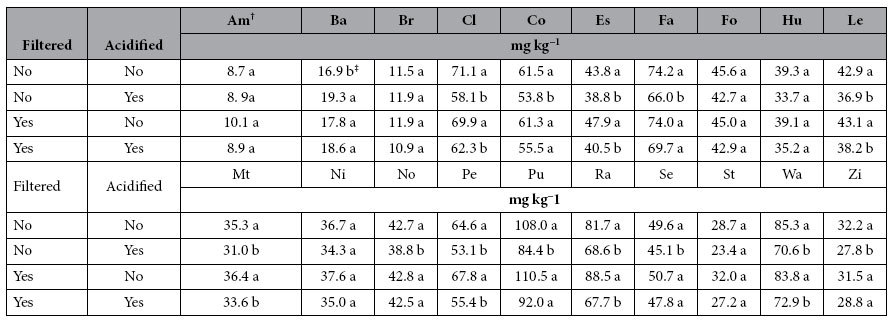
Effects of filtration and acidification on total P concentration in the undigested NaHCO_3_ fraction of soil samples determined by ICP-OES.

^†^Soil series are: Am: Amarillo, Ba: Barnes, Br: Brazil, Cl: Clarion, Co: Cordova, Es: Esthervile, Fa: Fargo, Fo: Formdale, Hu: Hubbard, Le: Lester, Mt: Mt. Carroll, Ni: Nicollet, No: Normania, Pe: Pella, Pu: Pullman, Ra: Randall, Se: Seaton, St: Storden, Wa: Walter, Zi: Zimmerman.

^‡^Means for a soil series followed by the different letters within filtration and dilution are significantly different (*P* < 0.05).

**Table 5:**
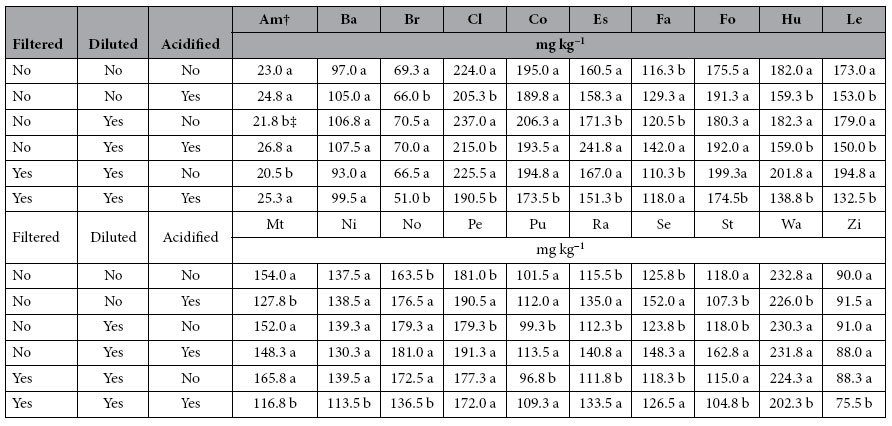
Effects of dilution and acidification on total P concentration in the undigested NaOH fraction of soil samples determined by ICP-OES.

^†^Soil series are: Am: Amarillo, Ba: Barnes, Br: Brazil, Cl: Clarion, Co: Cordova, Es: Esthervile, Fa: Fargo, Fo: Formdale, Hu: Hubbard, Le: Lester, Mt: Mt. Carroll, Ni: Nicollet, No: Normania, Pe: Pella, Pu: Pullman, Ra: Randall, Se: Seaton, St: Storden, Wa: Walter, Zi: Zimmerman.

^‡^Means for a soil series followed by the different letters within filtration and dilution are significantly different (*P* < 0.05).

**Table 6:** Total P concentration in the sequentially extracted HCl fraction of soil samples determined by ICP-OES after undigested, block digestion, and autoclave digestion treatments.

Soil Series	Filtered	Undigested		Block digestion		Autoclave digestion	
		mg kg^−1^					
Amarillo	No	22.3	a†	23.1	a	14.8	c
	Yes	19.3	b	18.1	b	14.8	c
Barnes	No	95.3	b	112.3	a	94.5	b
	Yes	98.0	b	60.0	c	102.3	b
Brazil^‡^	No	.		.		.	
	Yes	.		.		.	
Clarion	No	54.5	cd	77.8	a	57.3	c
	Yes	56.3	c	52.3	d	60.3	b
Cordova	No	185.8	c	235.3	a	187.0	c
	Yes	189.8	c	161.5	d	217.8	b
Estherville	No	96.5	b	79.3	c	105.5	a
	Yes	98.3	b	68.0	d	98.3	b
Fargo	No	363.0	c	415.0	b	459.3	a
	Yes	362.0	c	281.3	d	409.3	b
Formdale	No	107.3	c	136.5	a	117.0	b
	Yes	110.3	c	94.5	d	97.5	d
Hubbard	No	38.8	c	55.3	a	43.3	b
	Yes	39.5	c	34.8	d	34.8	d
Lester	No	48.8	c	67.8	a	59.3	b
	Yes	49.3	c	42.0	d	47.5	c
MtCarrol	No	46.3	c	61.0	a	53.0	b
	Yes	44.0	c	37.5	d	45.0	c
Nicollet	No	25.0	c	35.3	a	30.0	b
	Yes	21.5	c	16.0	d	21.3	c
Normania	No	51.3	b	48.0	c	47.5	c
	Yes	51.5	b	6.8	d	62.3	a
Pella	No	33.5	ab	28.0	c	29.3	bc
	Yes	35.0	a	5.0	d	35.8	a
Pullman	No	130.8	b	114.5	c	146.5	a
Yes	118.5	c	91.0	d	135.3	b	
Randall	No	254.3	b	209.8	d	265.0	ab
	Yes	235.0	c	258.0	b	272.8	a
Seaton	No	81.5	b	78.8	b	66.8	c
	Yes	81.3	b	9.8	d	95.5	a
Storden	No	35.3	bc	37.0	b	37.8	b
	Yes	30.0	c	44.3	a	31.0	c
Walter	No	92.3	ab	86.8	bc	80.3	d
	Yes	96.8	a	66.0	e	82.5	cd
Zimmerman	No	81.5	a	79.8	a	67.8	b
	Yes	83.8	a	57.5	c	83.5	a

^†^Means for a soil series followed by the different letter are significantly different (*P* < 0.05).

^‡^No measurable HCl P was detected in this soil.

**Table 7:** Average Effects of treatments on total organic P (P_o_) concentration in the four sequentially extracted fractions of soil samples calculated from the difference in P concentration in undigested, block digested and autoclave digested extracts determined by ICP-OES and blue colorimetry.

Soil Series	Filtered	Undigested		Block digestion		Autoclave digestion	
		mg kg^−1^					
Amarillo	No	30.0	b	55.3	ab	32.3	b
	Yes	26.3	b	79.3	a	17.3	b
Barnes	No	101.0	bc	188.5	a	135.5	b
	Yes	85.3	c	83.0	c	94.5	bc
Brazil	No	68.5	cd	127.5	a	73.3	c
	Yes	58.0	de	97.5	b	54.8	e
Clarion	No	220.5	b	282.3	a	233.3	b
	Yes	195.8	c	192.5	c	176.0	d
Cordova	No	239.0	c	348.3	a	274.8	b
	Yes	216.5	d	182.0	e	224.0	cd
Estherville	No	172.3	a	160.8	a	170.3	a
	Yes	143.5	a	76.5	b	168.0	a
Fargo	No	221.3	b	308.3	a	346.0	a
	Yes	209.0	b	155.3	c	236.8	b
Formdale	No	172.3	ab	175.0	ab	160.5	b
	Yes	188.3	a	159.5	b	173.0	ab
Hubbard	No	186.8	b	158.5	c	207.5	a
	Yes	207.5	a	175.3	b	183.5	b
Lester	No	168.3	bc	160.5	bc	216.0	a
	Yes	173.5	b	149.0	c	162.3	bc
MtCarroll	No	151.5	bc	161.5	bc	183.3	a
	Yes	170.8	ab	143.5	c	147.5	c
Nicollet	No	115.8	c	147.8	a	152.8	a
	Yes	119.0	c	119.5	c	135.3	b
Normania	No	155.8	ab	159.5	a	127.8	c
	Yes	139.3	bc	97.8	d	170.8	a
Pella	No	138.3	ab	104.0	b	140.8	a
	Yes	137.0	ab	158.8	a	42.0	c
Pullman	No	172.3	a	100.5	c	188.8	a
	Yes	147.5	b	36.5	d	138.8	b
Randall	No	158.3	a	84.3	b	173.0	a
	Yes	165.8	a	60.7	b	169.0	a
Seaton	No	116.0	a	107.0	ab	88.5	c
	Yes	97.3	bc	30.8	d	111.5	a
Storden	No	113.0	b	125.5	ab	108.3	bc
	Yes	114.0	b	154.5	a	71.0	c
Walter	No	215.0	a	156.5	b	168.8	b
	Yes	204.3	a	154.3	b	142.8	b
Zimmerman	No	109.5	b	102.5	b	84.8	b
	Yes	104.0	b	217.5	a	103.3	b

^†^Means for a soil series followed by the different letter are significantly different (*P* < 0.05).
